# Real-time PCR Demonstrates *Ancylostoma duodenale* Is a Key Factor in the Etiology of Severe Anemia and Iron Deficiency in Malawian Pre-school Children

**DOI:** 10.1371/journal.pntd.0001555

**Published:** 2012-03-06

**Authors:** Femkje A. M. Jonker, Job C. J. Calis, Kamija Phiri, Eric A. T. Brienen, Harriet Khoffi, Bernard J. Brabin, Jaco J. Verweij, Michael Boele van Hensbroek, Lisette van Lieshout

**Affiliations:** 1 Global Child Health Group, Emma Children's Hospital, Academic Medical Centre, Amsterdam, The Netherlands; 2 Community Health Department, College of Medicine, Blantyre, Malawi; 3 Department of Parasitology, Leiden University Medical Center, Leiden, The Netherlands; George Washington University Medical Center, United States of America

## Abstract

**Background:**

Hookworm infections are an important cause of (severe) anemia and iron deficiency in children in the tropics. Type of hookworm species (*Ancylostoma duodenale* or *Necator americanus*) and infection load are considered associated with disease burden, although these parameters are rarely assessed due to limitations of currently used diagnostic methods. Using multiplex real-time PCR, we evaluated hookworm species-specific prevalence, infection load and their contribution towards severe anemia and iron deficiency in pre-school children in Malawi.

**Methodology and Findings:**

*A. duodenale* and *N. americanus* DNA loads were determined in 830 fecal samples of pre-school children participating in a case control study investigating severe anemia. Using multiplex real-time PCR, hookworm infections were found in 34.1% of the severely anemic cases and in 27.0% of the non-severely anemic controls (p<0.05) whereas a 5.6% hookworm prevalence was detected by microscopy. Prevalence of *A. duodenale* and *N. americanus* was 26.1% and 4.9% respectively. Moderate and high load *A. duodenale* infections were positively associated with severe anemia (adjusted odds ratio: 2.49 (95%CI 1.16–5.33) and 9.04 (95%CI 2.52–32.47) respectively). Iron deficiency (assessed through bone marrow examination) was positively associated with intensity of *A. duodenale* infection (adjusted odds ratio: 3.63 (95%CI 1.18–11.20); 16.98 (95%CI 3.88–74.35) and 44.91 (95%CI 5.23–385.77) for low, moderate and high load respectively).

**Conclusions/Significance:**

This is the first report assessing the association of hookworm load and species differentiation with severe anemia and bone marrow iron deficiency. By revealing a much higher than expected prevalence of *A. duodenale* and its significant and load-dependent association with severe anemia and iron deficiency in pre-school children in Malawi, we demonstrated the need for quantitative and species-specific screening of hookworm infections. Multiplex real-time PCR is a powerful diagnostic tool for public health research to combat (severe) anemia and iron deficiency in children living in resource poor settings.

## Author Summary

Hookworm infections are a major cause of childhood anemia and iron deficiency. Two hookworm species exist of which *Ancylostoma duodenale* is the less common, yet causing more blood loss than *Necator americanus*. Although species differentiation and quantification are both of clinical importance, these are often not performed as the technique is complex and laborious using microscopy. Multiplex real-time PCR is a novel diagnostic tool which allows hookworm species differentiation and infection quantification. We applied this test in 830 stool samples of Malawian children with and without severe anemia. The prevalence of hookworm infections was high. *A. duodenale* was unexpectedly more prevalent than *N. americanus*. *A. duodenale* infections were associated with increased risk for severe anemia and iron deficiency, both of which increased with infection load. The study identifies a need for the quantitative screening of species-specific hookworm infections, which readily can be achieved by real-time-PCR. *A. duodenale* was independently associated with severe anemia and iron deficiency in our study population.

## Introduction

*Ancylostoma duodenale* and *Necator americanus* are soil transmitted nematodes responsible for an estimated 576–740 million human hookworm infections worldwide [1]–[3]. Hookworm infection often leads to anemia and iron deficiency, major causes of sickness and delayed cognitive development, especially in pre-school children [4]. Hookworm, therefore, is one of the most important infections in terms of disease burden in developing countries [5]–[7] with a major impact on public health [8]–[11].

The intensity of the hookworm infection is related to the severity of disease [5], [10]. As adult hookworms attach to and feed from the bowel mucosa of the infected host, they are the direct cause of intestinal blood loss, which often gives rise to iron deficiency and anemia. Crompton and Whitehead proposed a model describing the relationship between the actual number of adult worms present in the intestines, and the hosts iron status; increased infection load was associated with lower iron levels [12], [13]. The conventional method for determination of infection load is done with a proxy marker, number of eggs, using Kato-Katz microscopy slides with a fixed amount of feces [14]. However, although useful for estimating prevalence in highly endemic areas, the use of Kato-Katz microscopy for estimating intensity is laborious and frequently omitted.

*N. americanus* and *A. duodenale* are considered to have distinct geographical distribution; *N. americanus* is predominant in tropical environments, whereas *A. duodenale* is adapted to colder and drier circumstances [15], [16]. Based on studies of experimental human hookworm infections using labeled erythrocytes, daily blood loss is estimated to range from 0.03 to 0.30 ml per worm per day, with *A. duodenale* causing 2 to 10 times more blood loss per worm than *N. americanus* [12], [13]. This is consistent with a few detailed clinical studies which indicated a stronger association of *A. duodenale* infection with anemia than *N. americanus* [10], [15], [17]. Due to the complexity of conventional diagnostic procedures (microscopy by a skilled technician after 7 days of stool culture), differentiation of hookworm species rarely has been done in population-based surveys [18], [19]. This has been justified on the basis that prevalence of *A. duodenale* infection was limited and therefore the contribution to global disease burden was low, despite causing higher blood loss [15], [20]. Furthermore, as both species respond to the same anthelmintic treatment, differentiation is not considered essential for treatment. Yet post-treatment data is scarce and extremely little is known about the species-specific effects of mass drug administration [10], [21].

An alternative diagnostic procedure is multiplex real-time PCR which allows species-specific identification, as well as quantification of parasite DNA in human stool samples; based on selected targets and tested on a panel of well-defined DNA and stool samples, these assays were found to be 100% specific and substantially more sensitive compared with microscopy [22]–[25]. Through the use of different fluorescent labels in a closed system, multiple targets can be detected simultaneously within a single reaction tube with a low risk of contamination, which is ideal for high-throughput analysis. These assays offer significant potential for large scale population based surveys.

The objective of this study was, using a species-specific multiplex real-time PCR, to determine prevalence of the two hookworm species, infection load and species association with severe anemia and bone marrow iron deficiency in pre-school Malawian children.

## Methods

### Study design and study population

The study was part of a large case-control study on severe anemia in pre-school Malawian children (Figure S1). A detailed description of the study has been previously published elsewhere [26], [27]. In brief three groups of children were recruited between 2002 and 2004 in an urban and rural setting in Southern Malawi. Cases were children (aged 6–60 months) presenting with severe anemia (hemoglobin<5.0 gram per decilitre). For each case, two control children were enrolled, a community control living within 100 to 1000 meters of the case, and a hospital control, presenting at the same hospital or outpatient facility as the case. Controls were eligible for recruitment if aged 6–60 months and if their hemoglobin level was at least 5.0 gram per decilitre.

### Ethics statement

The study was approved by the Ethics Committees of the College of Medicine, University of Malawi, and the Liverpool School of Tropical Medicine, United Kingdom. Written informed consent was obtained from the parent or guardian.

### Laboratory investigations

Hemoglobin (Hb) concentrations measured by HemoCue B-Hemoglobin analyzer (HemoCue, Ängelholm, Sweden) were used to assess eligibility for the study; results of full blood count analyses by Coulter counter analyzer (Beckman Coulter, Durban, South Africa) were used for statistical analyses. In cases only, a bone marrow sample was collected, and slides were stained with Hematognost Fe (Merck, Darmstadt, Germany) and graded for iron content [28]. Bone marrow examination was performed as part of the main study to investigate etiology of severe anemia. Bone marrow smears were assessed by a histological grading method which classifies iron status into six grades, iron deficiency was defined as a bone marrow smear score of 0 or 1 [29]. In all study participants a stool sample was collected for parasitological examination. In order not to delay required treatment in case of hookworm infection, stool samples were examined for ova by microscopy of direct smears and a single 25 mg Kato-slide [30], [31]. The slides were read within 30 minutes by well trained microscopists and the number of hookworm eggs and presence of other helminth infections were recorded. An aliquot (approximately 0.5 gram) was stored at −20°C for later DNA isolation which was performed in a separate room at the research laboratory in Malawi. PCR was performed in Leiden University, the Netherlands. To prevent contamination with PCR products every step of the procedure was performed in separate rooms. DNA was isolated using feces suspensions of 200 µl (±0.5 gram of stool per ml PBS containing 2% poly-vinyl-poly-pyrolidone (Sigma, Steinheim, Germany)) and heated for 10 minutes at 100°C. After sodium-dodecyl-sulphate-proteinase K treatment (overnight at 55°C), DNA was isolated with QIAampTissue Kit spin columns (QIAgen, Hilden, Germany) [32]. Extracted DNA samples were transported for multiplex real-time PCR assessment in the Netherlands. For detection of inhibition of the amplification process, DNA of Phocin Herpes Virus 1 (PhHV-1) was added to each PCR mixture as an internal control and detected by PhHV-1 specific primers and probe [33]. *A. duodenale* and *N. americanus* multiplex real-time PCR was performed as described previously; targeting the Internal Transcribed Spacer 2 (ITS-2) sequence of each species and using minor groove binding detection probes [25]. The ITS-2 is a conserved DNA target part of the whole genome present in all life stages. In brief, amplification reactions were performed in a volume of 25 µl with PCR buffer (HotstarTaq master mix, QIAgen, Germany), 5 mM MgCl_2_, 2.5 µgram Bovine Serum Albumin (Roche Diagnostics Nederland B.V., Almere, the Netherlands), 1.5 pmol of each *A. duodenale*-specific primer, 2.5 pmol of NED-labeled *A. duodenale*-specific MGB-Taqman probe (Applied Biosystems, Warrington, U.K.), 5 pmol of each *N. americanus*-specific primer, 2.5 pmol of FAM-labeled *N. americanus*-specific MGB-Taqman probe (Applied Biosystems, Warrington, U.K.), 3.75 pmol of each PhHV-1-specific primer, and 2.5 pmol of PhHV-1-specific Cy5-double-labeled probe (Biolegio, Nijmegen, The Netherlands) and 5 µl of the DNA sample. Amplification consisted of 15 min at 95°C followed by 50 cycles of 15 s at 95°C and 60 s at 60°C. Negative and positive controls were included in each amplification-run. Amplification, detection and data analysis was performed with the AB7500 real-time PCR system (Applied Biosystems, Warrington, U.K.). The cycle threshold (Ct), meaning the amplification cycle in which the level of fluorescent signal exceeds background fluorescence, was used as the PCR output, reflecting parasite species-specific DNA load in the stool samples tested. Hookworm intensity categories were defined as following: low (Ct>35.0 and<50), moderate (Ct>25.0 and ≤35.0) or high (Ct≤25.0). None of the PCR analyzed samples were excluded due to inhibition as all runs showed a Ct-value for the internal PhHV-1 control within the expected range.

### Statistical methods

Data were analyzed with the use of STATA 9 (Stata Corporation, TX) and PASW Statistics 18 (SPSS, Chicago, Illinois) statistical computer packages. Cross-sectional analyses were completed to assess the correlation of severe anemia and iron deficiency with PCR detected hookworm load per species. Using Chi-square test hookworm prevalence per species was compared individually across severely anemic and non-severely anemic as well across iron deficient and iron replete study groups. Spearman's rank correlation was used for calculation of concordance between Ct-values with hemoglobin and number of iron fragments in the bone marrow. The combined association of characteristics related to risk of severe anemia and iron deficiency was examined by two multivariate logistic regression models, correcting for potential confounding factors. Potential etiologies of severe anemia were entered in the model if they were associated with severe anemia in univariate analysis (p<0.10). The model was adjusted for subjects' baseline characteristics (age, sex) and other potential confounders: recent hematinic or anti-malarial treatment, history of transfusions and death of a parent [22]. With this model we compared all cases with the two control groups combined, stratified by study location. To explore the possibility that different patient characteristics were important in the two control groups, separate analyses were performed with the community and hospital control groups. To specify the association between iron deficiency (dependent variable) and hookworm (independent variable) we adjusted for subjects' baseline characteristics (age, sex, study location) and other potential confounders (HIV infection and wasting (defined as a Z-score of weight for height <−2 [34])). Stepwise backward multiple logistic regression analyses were performed; *p* values less than 0.05 were considered as statistically significant. The latter analyses only included case patients with bone marrow aspirate results. For both analyses attributable-risks were calculated using adjusted odds ratios [35]. When both hookworm species were analyzed together, the lowest Ct-value was counted. Reported *p*-values are two-sided.

## Results

Stool samples of 830 (72.9%) of the 1138 children enrolled in the severe anemia study were stored for real-time PCR analysis (Figure S1). Table 1 summarizes mean hemoglobin and the general characteristics per study group (see also table S2). The mean age was lower in the case group; for which we adjusted in the multivariate model. Other baseline characteristics were not statistically different. Bone marrow iron examination was performed in a subgroup of 160 severely anemic children (cases) (Table 2, S1). Baseline characteristics were not significantly different between children with or without available stool sample or bone marrow sample (data not shown).

**Table 1:** Baseline characteristics of 830 hookworm-PCR tested children stratified per study group.

	Cases	Hospital Controls	Community Controls
Characteristic	Hb≤5.0 g/dL (N = 252)	Hb>5.0 g/dL (N = 291)	Hb>5.0 g/dL (N = 287)
Living in an urban area	134 (53.2%)	150 (51.5%)	146 (50.9%)
Male	119/252 (47.2%)	147/291 (50.5%)	139/287 (48.4%)
Age in months (mean ± SD)	19.9±12.6	22.7±12.0	25.6±13.6
Hemoglobn* in g/dL (mean ± SD)	3.6±0.8	9.6±2.2	9.9±1.9

* n = 826.

**Table 2:** Baseline characteristics of sub population (severely anemic cases) stratified per iron status.

	Iron deficient†	Iron replete§
Characteristic	(n = 68)	(n = 92)
Living in an urban area	45 (66.2%)	52 (56.5%)
Male	25 (36.8%)	49 (53.2%)
Age in months (mean ± SD)	18.1±10.4	21.6±13.8
Iron fragments (mean ± SD)	0.3±0.5	3.8±1.0

† Iron deficiency was defined as a bone marrow iron grade of none (grade 0) or very slight (grade 1).

§ Iron replete means sufficient iron (≥grade 2).

### Hookworm

Microscopy was done on 780 (94.0%) of the 830 stool samples. Failure was due to small sample stool volume or constitution (too watery for Kato-slide examination). Hookworm eggs were identified in 44 of 780 (5.6%) samples, of which 18 (37.5%) showed a high-load infection, defined by more than 1.000 eggs per gram feces (epg). In the severely anemic children (cases), significantly more high-load hookworm infections were detected with microscopy compared to the controls; 5.9% (14/236) vs. 0.7% (4/544), *p*<0.001). Real-time PCR identified 242 (29.2%) hookworm infections in the 830 children. *A. duodenale* and *N. americanus* DNA was detected in 217 (26.1%) and 41 (4.9%) children, respectively. Six children were infected with both species. Within the healthy study population (community controls) 73 hookworm infections were detected (25.4%) of which the larger part was caused by *A. duodenale* (Table 3). In 182 (83.5%) of the 218 PCR positive samples tested parasitologically, microscopy did not identify the hookworm infection, 130 (71.4%) of these samples showed low quantities of hookworm DNA (Ct>35). Furthermore in 8 (18.2%) of the 44 microscopy positive samples the presence of hookworm was not confirmed with real-time PCR. These 8 samples had a median of 220 eggs per gram.

**Table 3:** PCR-determined hookworm distribution stratified per study group.

		Cases	Hospital Controls	Community Controls
		Hb≤5.0 (N = 252)	Hb>5.0 (N = 291)	Hb>5.0 (N = 287)
Any hookworm infection		86 (34.1%) *	83(28.5%)	73(25.4%)
A. duodenale	positive	81(32.1%) **	72(24.7%)	64(22.3%)
Infection load	low	32 (12.7%)	54 (18.6%)	46 (16.0%)
	moderate	27 (10.7%) **	15 (5.2%)	17 (5.9%)
	high	22 (8.7%) ***	3 (1.0%)	1 (0.3%)
N. americanus	positive	10 (4.0%)	19(6.5%)	12(4.2%)
Infection load	low	6 (2.4%)	14 (4.8%)	7 (2.4%)
	moderate	3 (1.2%)	3 (1.0%)	5 (1.7%)
	high	1 (0.4%)	2 (0.7%)	0 (0%)

Infection load is defined by the following cycle thresholds (Ct): low 35<Ct<50; moderate 25<Ct≤35; high Ct≤25. In case of dual infection the lowest Ct-value was counted. Cases are compared with combined control groups using Chi-square.

* P<0.05.

** P<0.01;

*** P<0.001.

### Anemia

**Figure 1:**
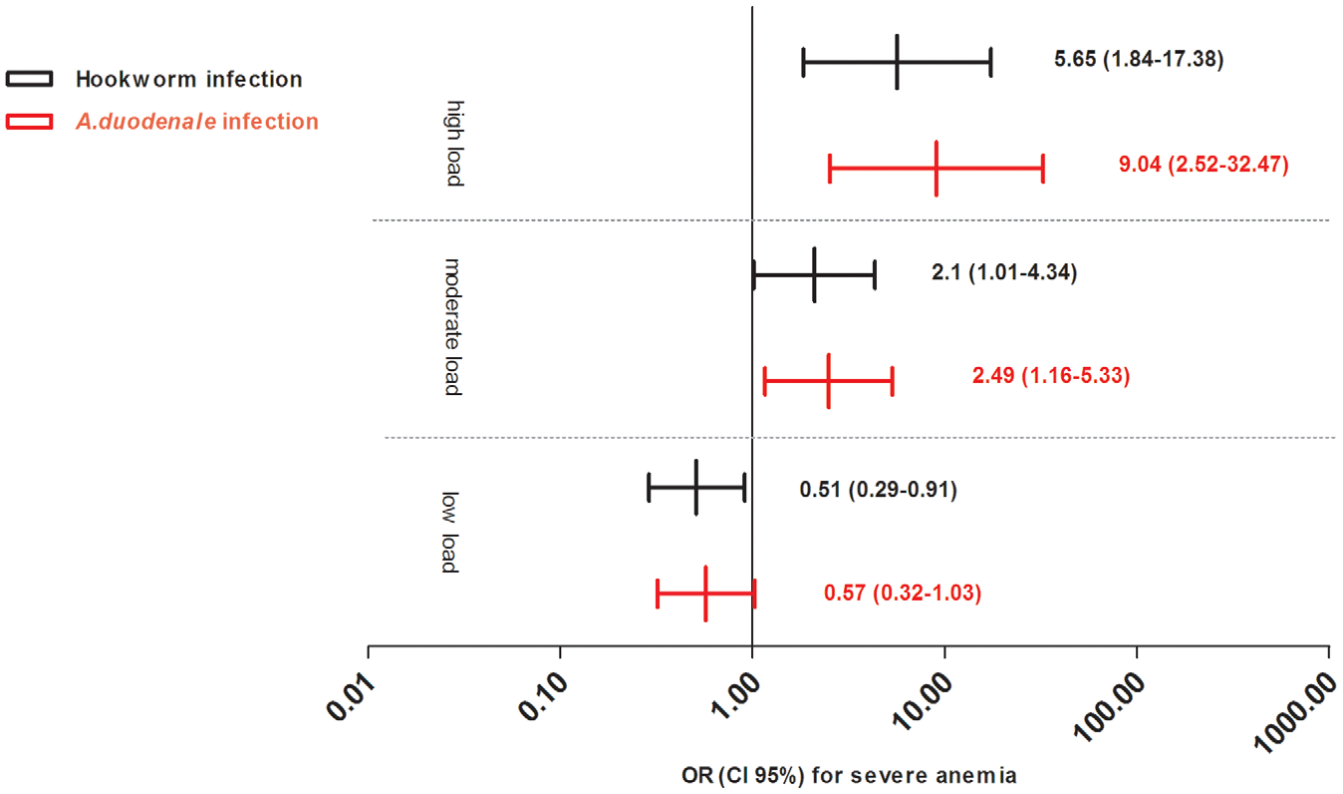
PCR-detected hookworm infection and its association with severe anemia. Displayed are the adjusted Odds Ratios and 95% confidence intervals for hookworm infection and its association with severe anemia. Hookworm infection is defined as an *A. duodenale* and/or *a N. americanus* infection; infection load is defined by the following cycle thresholds (Ct): low 35<Ct<50; moderate 25<Ct≤35; high Ct≤25. In case of dual infection the lowest Ct-value was counted. Severe anemia is defined as a hemoglobin of <5.0 g per decilitre. The multivariate model was adjusted for age, sex, recent use of hematinics or anti-malarial treatment, history of transfusions, death of a parent, limited maternal education (mother did not attend secondary school), wasting (defined as a Z-score of weight for height <−2), vitamin B12 deficiency (<20 ng/dL), vitamin A deficiency (<20 ug/dL), HIV, Epstein-Barr virus, bacteremia, malaria parasitemia, G6PD deficiency and IL-10-23 mutations.

Hookworm infections were detected with PCR in 34.1% of the severely anemic cases and in 27.0% of the non-severely anemic controls (*p*<0.05). *A. duodenale* was found in 32.1% of the severely anemic cases and in 23.5% of the non-severely anemic controls (*p*<0.01, Table 3). Also the prevalence of high intensity infections (Ct<25) of *A. duodenale* infections was different; 8.7% among cases and 0.7% among controls (*p*<0.001). Additionally, in moderately anemic children (Hb 5–11 g/L) prevalence of *A. duodenale* was higher, 26.0% (105/404), than in non-anemic children (Hb≥11 g/dL), 17.6% (30/170) (*p*<0.03). There was no difference in prevalence of *N. americanus* infections between moderately anemic and non-anemic children. Within the whole study population (cases and controls combined) hookworm infection load and hemoglobin were negatively correlated (spearman correlation coefficient ρ = 0.176, *p*<0.01, n = 826), which was mainly due to *A. duodenale* (ρ = 0.173, *p*<0.01, n = 826) and not to *N. americanus* (ρ = 0.042, *p* = 0.2, n = 826). After correction for other causal factors of severe anemia using multivariate analysis, *A. duodenale* remained a significant risk factor for severe anemia; odds ratios increased with infection load and are shown in figure 1 and table S3.

### Iron deficiency

**Figure 2:**
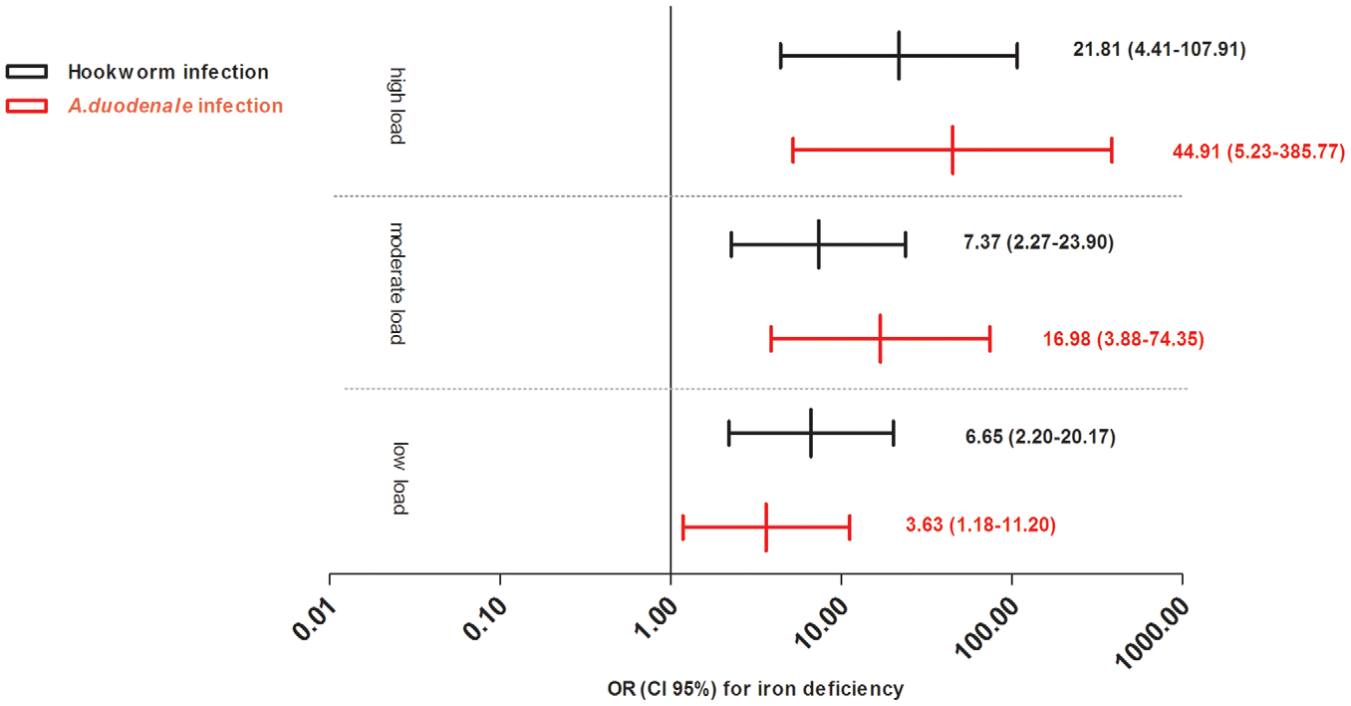
PCR-detected hookworm infection and its association with iron deficiency. Displayed are the adjusted Odds Ratios and 95% confidence intervals for hookworm infection and its association with iron deficiency. Hookworm infection is defined as an *A. duodenale* and/or *a N. americanus* infection; infection load is defined by the following cycle thresholds (Ct): low 35<Ct<50; moderate 25<Ct≤35; high Ct≤25. In case of dual infection the lowest Ct-value was counted. Iron deficiency is defined as a bone marrow smear score of 0 or 1 [29]. The multivariate model was adjusted for age, sex, study location, HIV (human immunodeficiency virus) infection and wasting (defined as a Z-score of weight for height <−2). These analyses include only children with severe anemia (hemoglobin of <5.0 g per decilitre).

Iron deficiency was prevalent in 42.5% (68/160) of severely anemic children who had available bone marrow samples. Of the iron deficient children 60.3% (41/68) had a hookworm infection, compared to 16.3% (15/92) for the iron replete group (*p*<0.0001). This difference in prevalence was only noted for *A. duodenale* infections and was greater in children with a high infection load (Table 4). Infection load for *A. duodenale*, but not for *N. americanus*, was negatively correlated with the fragmental iron staining score (spearman correlation coefficient ρ = 0.536, *p*<0.01 and ρ = 0.015, *p* = 0.9 respectively, n = 160). The association between hookworm infection load and iron deficiency remained significant after correcting for other factors using multivariate analysis (Figure 2, table S4).

**Table 4:** PCR-determined hookworm distribution in severely anemic children stratified per iron status.

		Iron deficient†	Iron replete§
		(n = 68)	(n = 92)
**Any hookworm infection**		**41 (60.3%)** ***	**15 (16.3%)**
***A. duodenale***	**positive**	**39 (57.4%)** ***	**12 (13.0%)**
Infection load	low	12 (17.6%)	8 (8.7%)
	moderate	13 (19.1%)**	3 (3.3%)
	high	14 (20.6%)***	1 (1.1%)
***N. americanus***	**positive**	**5 (7.4%)**	**4 (4,3%)**
Infection load	low	0 (0.0%)	2 (2.1%)
	moderate	1 (1.5**%**)	1 (1.1**%**)
	high	4 (5.9**%**)	1 (1.1**%**)

Infection load is defined by the following cycle thresholds (Ct): low 35<Ct<50; moderate 25<Ct≤35; high Ct≤25. In case of dual infection the lowest Ct-value was counted. Cases are compared with combined control groups using Chi-square.

** P<0.01;

*** P<0.001.

† Iron deficiency was defined as a bone marrow iron grade of none (grade 0) or very slight (grade 1).

§ Iron replete means sufficient iron (≥grade 2).

## Discussion

Using the multiplex real-time PCR test for hookworm identification we revealed the hidden burden of hookworm in pre-school children in Southern Malawi; hookworm prevalence was much higher than expected. We have shown that hookworm infections, mainly *A. duodenale*, are significantly associated with the development of iron deficiency and severe anemia in pre-school children in Malawi. One of the strengths of this study is the sample size and comprehensive data set. It is the first report assessing the association of hookworm intensity and species differentiation with iron deficiency measured with the reference standard for iron status.

Severe anemia is a leading cause of hospital admissions and death in children living in sub-Saharan Africa, where between 12–29% of all children admitted to hospital are severely anemic and require blood transfusion. This results in high mortality (8–17%) [36]–[38]. The cause of severe anemia however, often remains unexplained. Using the same dataset, we have previously reported factors associated with severe anemia and identified hookworm infection, diagnosed by microscopy, as a significant correlate of infection [26]. This applied to infections with a high load (>1.000 epg) only. Using real-time PCR we now demonstrate that high-load infections have the greatest impact on disease burden, however also moderate *A. duodenale* infections were associated with significantly increased risk of severe anemia. This indicates that the association of microscopy-detected hookworm with severe anemia is an underestimation of the real impact of hookworm infections [39]. The disease burden of *A. duodenale* infections may be a significant contributor to unexplained severe anemia prevalence in sub-Saharan Africa.

Iron deficiency is often considered the same as anemia, especially in resource poor settings where both are very common. Estimations of prevalence of iron deficiency in Malawi vary from 20–40% [40]. Yet, iron deficiency without anemia is important to recognize as it may delay cognitive development in pre-school and school-age children [6], [7], [41]. On the other hand, anemia of inflammation is often anemia without iron deficiency. During inflammation iron supplementation should be avoided, since the absorption will be poorly and may possibly increase the risk of infection [42]–[46]. Thus in stead of presumtive treatment of iron deficiency, prevention of iron deficiency would be rather preferred and should include the use of anthelmintics if prevalence of *A. duodenale* is as high as in this study.

Unexpected was the high prevalence of *A. duodenale* whilst we expected *N. americanus* to be the dominant specie in this area [15], [16]. This is important as we showed that *A. duodenale* was an independent risk factor for both moderate/severe anemia and iron deficiency. Based on the results from ‘healthy’ community controls, this study suggests that *A. duodenale* was the predominant hookworm species in children below five years of age in this area in southern Malawi. Whether this pattern is similar for all ages needs to be investigated, but PCR based analysis of stool samples from a community-based cross-sectional survey in the nearby Mozambique indicated that the ratio between the two hookworm species changes with age; *A. duodenale* was the predominant species in children, whereas in adults both species were almost equally present (Van Lieshout, unpublished data). Clearly more studies are needed to determine the species specific distribution and their risk factors in different regions, and how they relate to the burden of infection.

A surprising finding was that severe anemia was less common in children having low-load hookworm infections (35<Ct<50) when compared to non-infected children (Figure 1). We considered two possible explanations. Firstly, low-load hookworm infections cause iron deficiency which protects against bacteremia, a cause of severe anemia [26]. Alternatively a low hookworm load is seen in children with an effective immune response which are able to control their hookworm infection and other infections that may cause severe anemia. The proportional benefit of treating low-load hookworm infections requires further study as even these infections may contribute to burden of disease [8].

From a public health perspective the implication of this diagnostic method could be substantial as hookworm prevalence based on conventional microscopy seems largely underestimated, with the consequence that substantial areas may unjustly remain untreated. Screening for hookworm with real-time PCR would lead to more reliable prevalence data which should benefit the efficiency of mass drug administration. In 2007 the World Health Organization stated that in areas with a hookworm prevalence of more than 20%, all pre-school and school-age children should yearly be treated with anthelmintics, and where prevalence is more than 50% they should be treated twice a year [47]. Although large scale deworming has been proven to decrease hookworm prevalence and contribute to an improved health and well-being [10], [48], the importance of appropriate interventions still remain neglected in most endemic countries [49] and there is some debate whether administration of anthelmintic drugs results in substantial improvement of hemoglobin concentration [21]. Differential effects of treatment might relate to geographic differences in species-specific distribution and infection load. Monitoring deworming programs using real-time PCR would provide more precise data on the effects of mass treatment. In addition, assessment of differences in treatment effects per species may diminish risk for the development of drug resistance.

A concern was that 18.2% (8/44) of the microscopy positives were PCR negative. This finding may indicate genetic variation of the PCR target gene [50]. On the other hand this was not supported by the fact that almost all PCR-missed infections showed low egg counts. Misidentification during microscopy could be another explanation. Nevertheless, most procedures have a certain chance to miss very light infections, and as examination is based on a small test sample volume only, it is probable that both eggs and free DNA are not completely homogeneously distributed within the stool sample. Hookworm was not detected by microscopy in 83.5% (182/218) of the PCR positive samples, since the specificity of real-time PCR was already proved to be close to 100% [25] this demonstrates the limited sensitivity of microscopy to detect hookworm infections even in an ideal laboratory setting. A limitation is that the association between iron deficiency and hookworm was only assessed in severely anemic children. These children may represent a small group of children with severe disease and interpretation of the associations may be different in the majority of otherwise “healthy children” infested with hookworm.

Although the value of real-time PCR for clinical diagnostics is limited in resource poor settings, this method brings forward new exciting prospects for epidemiology of intestinal parasites in these settings. The collection of stool samples in ethanol allows storage at room temperature and transportation to central research centers with facilities for real-time PCR. Moreover, using the same DNA isolation method simultaneously monitoring of other parasitic infections can be performed. For example *A. duodenale*, *N. americanus*, *Ascaris lumbricoides* and *Strongyloides stercoralis*-specific DNA can be detected in a single assay [51]–[53]. This all simplifies the complex organization of labor-intensive field studies in remote areas. An increasing number of research centers located within low income countries have access to real-time PCR technology. In combination with a trend of decreasing availability of well trained microscopists, real-time PCR is more recognized as an important diagnostic tool in research.

In conclusion, we have shown the need for quantitative screening of species-specific hookworm infections by demonstrating a much higher than previously expected prevalence of *A. duodenale*, and identified its significant contribution to severe anemia and bone marrow iron deficiency in pre-school children in Malawi. Multiplex real-time PCR is a powerful diagnostic tool in public health research to facilitate identification of the causes of (severe) anemia and iron deficiency in children living in resource poor settings.

## Supporting Information

Figure S1 **Flow chart study population.** This flowchart presents number of children enrolled in the main study, a case control study investigating etiology of severe anemia (I); number of children enrolled in the sub study investigating association of hookworm infection and severe anemia (II) and number of children enrolled in the sub study investigating association of hookworm infection and iron deficiency (III). HC: Hospital Control; CC: Community Control.
(DOC)

Table S1 **Additional baseline characteristics of 160 severe anaemic cases with available bone marrow stratified per iron status.**
(DOC)

Table S2 **Additional baseline characteristics of 830 hookworm-PCR tested children stratified per study location.**
(DOC)

Table S3 **Unadjusted and adjusted Odds ratios with 95% CI for severe anemia of all variables included in the models for hookworm infection (model I) and** ***A.duodenale*** **infection (model II).**
(DOC)

Table S4 **Unadjusted and adjusted Odds ratios with 95% CI for iron deficiency of all variables included in the models for hookworm infection (model I) and** ***A.duodenale*** **infection (model II).**
(DOC)

Checklist S1 **STROBE checklist.**
(DOCX)
