# Supplementary material for: Real-time PCR Demonstrates Ancylostoma duodenale Is a Key Factor in the Etiology of Severe Anemia and Iron Deficiency in Malawian Pre-school Children
Source: PLoS Negl Trop Dis. 2012 Mar 6;6(3):e1555. doi: 10.1371/journal.pntd.0001555 (PMC3295794; doi:10.1371/journal.pntd.0001555)
Supplement: Table S1 — Additional baseline characteristics of 160 severe anaemic cases with available bone marrow stratified per iron status. (DOC) [file pntd.0001555.s002.doc]

| **Table S1. Baseline characteristics of 160 severe anaemic cases with available bone marrow stratified per iron status** | | |
| --- | --- | --- |
|  | **Iron deficient †** | **Iron replete** |
| **Characteristic** | **(n=68)** | **(n=92)** |
| Living in an urban area | 45 (66.2 %) | 52 (56.5 %) |
| Male | 25 (36.8 %) | 49 (53.2 %) |
| Age in months (mean ± SD) | 18.1 ± 10.4 | 21.6 ± 13.8 |
| Iron fragments (mean ± SD) | 0.3 ± 0.5 | 3.8 ± 1.0 |
| HIV-infection | 10/65 (15.5%) | 7/87 (8.0%) |
| Wasting | 10/66 (15.2%) | 5/79 (6.3%) |
| † Iron deficiency was defined as marrow iron stores < 2 iron fragments. Iron replete means sufficient iron in marrow stores (≥ 2 iron fragments). Wasting is defined as a Z-score of weight for height < -2. | | |
