# Supplementary material for: Real-time PCR Demonstrates Ancylostoma duodenale Is a Key Factor in the Etiology of Severe Anemia and Iron Deficiency in Malawian Pre-school Children
Source: PLoS Negl Trop Dis. 2012 Mar 6;6(3):e1555. doi: 10.1371/journal.pntd.0001555 (PMC3295794; doi:10.1371/journal.pntd.0001555)
Supplement: Table S2 — Additional baseline characteristics of 830 hookworm-PCR tested children stratified per study location. (DOC) [file pntd.0001555.s003.doc]

| **Table S2 Baseline characteristics of 830 hookworm-PCR tested children stratified per study location** | | | | |  |
| --- | --- | --- | --- | --- | --- |
|  | **Cases** | **HC+CC combined** |  | **Hospital Controls** | **Community Controls** |
| **Characteristic** | Hb ≤5.0 g/dL (N=252) | Hb >5.0g/dL (N=578) |  | Hb >5.0 g/dL (N=291) | Hb >5.0g/dL (N=287) |
| Hemoglobin in g/dL (mean ± SD) (n=826) | 3.6 ± 0.8 | 9.7 (2.1) |  | 9.6 ± 2.2 | 9.9 ± 1.9 |
| Male | 119/252 (47.2 %) | 286/578 (49.5%) |  | 147/291 (50.5 %) | 139/287 (48.4 %) |
| Living in an urban area | 118/252 (46.8 %) | 282/578 (48.8%) |  | 150/291 (51.5 %) | 146/287 (50.9 %) |
| Age < 24 months | 177/252 (70.2%) | 323/578 (55.9%) |  | 178/291 (61.2%) | 145/187 (50.5%) |
| Recent hematinics | 50/252 (19.8%) | 29/576 (5.0%) |  | 16/290 (5.5%) | 13/286 (4.5 %) |
| Recent antimalarial treatment | 150/251 (59.8%) | 247/577 (47.5%) |  | 150/290 (51.7%) | 124/287 (43.2%) |
| History of transfusion | 35/252 (13.9%) | 30/578 (5.2%) |  | 17/291 (5.8%) | 13/287 (4.5%) |
| Death of a parent | 17/190 (8.9%) | 11/429 (2.6%) |  | 7/223 (3.1%) | 4/206 (1.9%) |
| Limited maternal education | 217/246 (88.2%) | 427/574 (74.4%) |  | 204/291 (70.1%) | 223/283 (78.9%) |
| Wasting | 29/229 (12.7%) | 34/533 (6.4%) |  | 24/261 (9.2%) | 10/272 (3.7%) |
| Vitamin B12 deficiency | 112/214 (52.3%) | 150/462 (32.5%) |  | 85/233 (36.5%) | 70/229 (30.6%) |
| Vitamin A deficiency | 164/177 (92.7%) | 126/201 (62.7%) |  | 71/92 (77.2%) | 55/109 (50.5%) |
| HIV-infection | 25/241 (10.4%) | 31/530 (5.8%) |  | 21/266 (7.6%) | 10/264 (3.8%) |
| Epstein-Barr virus | 65/179 (36.3%) | 80/435 (18.4%) |  | 42/221 (19.0%) | 38/214 (17.8%) |
| Bacteremia | 36/177 (20.3%) | 12/244 (4.9%) |  | 12/244 (4.9%) | not done |
| Malaria | 154/252 (61.1%) | 238/573 (41.7%) |  | 116/290 (41.0%) | 120/283 (42.4%) |
| G6PD deficiency | 30/220 (13.6%) | 37/454 (8.1%) |  | 15/230 (6.5%) | 22/224 (9.8%) |
| IL-10-23 mutations | 74/223 (33.2%) | 99/457 (21.7%) |  | 54/233 (23.2%) | 45/224 (20.1%) |

Recent use of hematinics or anti-malarial treatment: defined as in previous 4 weeks; limited maternal education: mother did not attend

secondary school; wasting: defined as a Z-score of weight for height < -2; vitamin B12 deficiency: < 20 ng /dL; vitamin A deficiency:< 20 ug /dL.
