# Supplementary material for: Real-time PCR Demonstrates Ancylostoma duodenale Is a Key Factor in the Etiology of Severe Anemia and Iron Deficiency in Malawian Pre-school Children
Source: PLoS Negl Trop Dis. 2012 Mar 6;6(3):e1555. doi: 10.1371/journal.pntd.0001555 (PMC3295794; doi:10.1371/journal.pntd.0001555)
Supplement: Table S3 — Unadjusted and adjusted Odds ratios with 95% CI for severe anemia of all variables included in the models for hookworm infection (model I) and A.duodenale infection (model II). (DOC) [file pntd.0001555.s004.doc]

| **Table S3 Unadjusted and adjusted Odds ratios with 95% CI for severe anemia of all variables included in the models for hookworm infection (model I) and *A.duodenale* infection (model II)** | | | | | | |
| --- | --- | --- | --- | --- | --- | --- |
| **SEVERE ANEMIA** | **unadjusted** | | **adjusted model I** | | **adjusted model II** | |
|  | **OR** | **95%CI** | **OR** | **95%CI** | **OR** | **95%CI** |
| male | 0,91 | 0,68-1,23 | 0,75 | 0.53-1.08 | 0,76 | 0.53-1.08 |
| age < 24 months, age continuous | 1,86 | 1,36-2,56 | 0,98 | 0.97-1.00 | 0,98 | 0,97-1,00 |
| recent hematinics | 3,90 | 2,40-6,35 | 3,00 | 1.81-4.96 | 2,94 | 1.78-4.87 |
| recent antimalarials | 1,23 | 0,91-1,66 | 1,72 | 1.19-2.49 | 1,73 | 1.20-2.51 |
| history of transfusions | 2,95 | 1,77-4,92 | 2,49 | 1.39-4.47 | 2,45 | 1.36-4.40 |
| death of a parent | 3,73 | 1,71-8,14 | 2,03 | 0.83-4.95 | 2,02 | 0.82-4.95 |
| limited maternal education | 2,09 | 1,36-3,20 | 2,61 | 1.58-4.32 | 2,60 | 1.57-4.31 |
| wasting | 2,13 | 1,26-3,59 | 1,70 | 0.96-3.04 | 1,70 | 0.95-3.04 |
| vitamin B12 deficiency | 2,28 | 1,64-3,18 | 2,21 | 1.38-3.55 | 2,18 | 1.36-3.50 |
| vitamin A deficiency | 7,51 | 3,99-14,14 | 2,65 | 1.25-5.60 | 2,68 | 1.26-5.71 |
| low load hookworm infection | 0.78 | 0.51-1.20 | 0,51 | 0.29-0.91 | ─ | |
| moderate load hookworm infection | 1.90 | 1.13-3.16 | 2,10 | 1.01-4.34 | ─ | |
| high load hookworm infection | 9.75 | 3.99-24.36 | 5,65 | 1.84-17.38 | ─ | |
| low load *a.duodenale*  infection | 0.83 | 0.54-1.28 | ─ | | 0,57 | 0.32-1.03 |
| moderate load *a.duodenale* infection | 2.19 | 1.27-3.75 | ─ | | 2,49 | 1.16-5.33 |
| high load *a.duodenale*  infection | 14.22 | 4.83-41.86 | ─ | | 9,04 | 2.52-32.47 |
| HIV | 1,86 | 1,07-3,23 | 1,90 | 0.97-3.73 | 1,96 | 1.00-3.84 |
| Epstein-bar virus | 2,53 | 1,71-3,73 | 1,75 | 1.10-2.79 | 1,78 | 1.12-2.83 |
| bacteremia | 4,94 | 2,49-9,80 | 5,09 | 2.43-10.65 | 5,16 | 2.46-10.79 |
| malaria parasitemia | 2,20 | 1,62-2,97 | 2,35 | 1.62-3.42 | 2,37 | 1.63-3.45 |
| G6PD deficiency | 1,78 | 1,07-2,97 | 2,31 | 1.25-4.29 | 2,30 | 1.24-4.28 |
| IL-10-23 mutations | 1,80 | 1,26-2,57 | 1,46 | 0.94-2.28 | 1,45 | 0.93-2.26 |

Recent use of hematinics or anti-malarial treatment: defined as in previous 4 weeks; limited maternal education: mother did not attend secondary school; wasting: defined as a Z-score of weight for height < -2; vitamin B12 deficiency: < 20 ng /dL; vitamin A deficiency :< 20 ug /dL; * age < 24 months for unadjusted model, age continuous for adjusted models. **Hookworm infection load is defined by the following cycle thresholds (Ct): low 35<Ct<50; moderate 25<Ct≤35; high Ct≤25. In case of dual infection the lowest Ct-value was counted.
