# Supplementary material for: Real-time PCR Demonstrates Ancylostoma duodenale Is a Key Factor in the Etiology of Severe Anemia and Iron Deficiency in Malawian Pre-school Children
Source: PLoS Negl Trop Dis. 2012 Mar 6;6(3):e1555. doi: 10.1371/journal.pntd.0001555 (PMC3295794; doi:10.1371/journal.pntd.0001555)
Supplement: Table S4 — Unadjusted and adjusted Odds ratios with 95% CI for iron deficiency of all variables included in the models for hookworm infection (model I) and A.duodenale infection (model II). (DOC) [file pntd.0001555.s005.doc]

| **Table S4 Unadjusted and adjusted Odds ratios with 95% CI for iron deficiency of all variables included in the models for hookworm infection (model I) and *A.duodenale* infection (model II).** | | | | | | |
| --- | --- | --- | --- | --- | --- | --- |
| **IRON DEFICIENCY** | **unadjusted** | | **adjusted model I** | | **adjusted model II** | |
|  | **OR** | **95%CI** | **OR** | **95%CI** | **OR** | **95%CI** |
| low load hookworm infection | 4.99 | 1.89-13.21 | 6.65 | 2.20-20.17 | − | |
| moderate load hookworm infection | 7.42 | 2.42-22.74 | 7.37 | 2.27-23.90 | − | |
| high load hookworm infection | 19.96 | 4.26-93.59 | 21.81 | 4.41-107.91 | − | |
| low load *a.duodenale*  infection | 4.14 | 1.54-11.14 | − | | 3.63 | 1.18-11.20 |
| moderate load *a.duodenale* infection | 11.95 | 3.18-44.99 | − | | 16.98 | 3.88-74.35 |
| high load *a.duodenale* infection | 38.62 | 4.86-306.91 | − | | 44.91 | 5.23-385.77 |
| age * | 0.98 | 0.95-1.00 | − | | 0.97 | 0.93-1.01 |
| male | 0.51 | 0.27-0.97 | 0.36 | 0.16-0.80 | 0.33 | 0.14-0.78 |
| living in an urban area | 0.39 | 0.21-0.75 | − | | − | |
| HIV | 2.1 | 0.75-5.80 | − | | 4.35 | 1.02-18.45 |
| wasting | 2.64 | 0.86-8.17 | − | | 3.12 | 0.80-12.15 |

Iron deficiency was defined as a bone marrow iron grade of none (grade 0) or very slight (grade 1). Hookworm infection load is defined by the following cycle thresholds (Ct): low 35<Ct<50; moderate 25<Ct≤35; high Ct≤25. In case of dual infection the lowest Ct-value was counted.* age < 24 months for unadjusted model, age continuous for adjusted models; wasting: defined as a Z-score of weight for height < -2.
