# Supplementary material for: Real-time PCR Demonstrates Ancylostoma duodenale Is a Key Factor in the Etiology of Severe Anemia and Iron Deficiency in Malawian Pre-school Children
Source: PLoS Negl Trop Dis. 2012 Mar 6;6(3):e1555. doi: 10.1371/journal.pntd.0001555 (PMC3295794; doi:10.1371/journal.pntd.0001555)
Supplement: Checklist S1 — STROBE checklist. (DOCX) [file pntd.0001555.s006.docx]

**STROBE Statement—checklist of items that should be included in reports of observational studies**

|  | Item No | Recommendation |
| --- | --- | --- |
| **Title and abstract** | 1 | (*a*) **Indicate the study’s design with a commonly used term in the title or the abstract**  page 1 |
|  |  | (*b*) **Provide in the abstract an informative and balanced summary of what was done and what was found**  page 1 |
| Introduction | | |
| Background/  rationale | 2 | **Explain the scientific background and rationale for the investigation being reported**  page 3 and 4 |
| Objectives | 3 | **State specific objectives, including any pre-specified hypotheses**  page 1, lines 20-21  page 2, lines 47-48  page 3, lines 77-79  page 3, lines 90-92  page 4, lines 111-113 |
| Methods | | |
| Study design | 4 | **Present key elements of study design early in the paper**  page 4, lines 117-125 |
| Setting | 5 | **Describe the setting, locations, and relevant dates, including periods of recruitment, exposure, follow-up, and data collection**  page 3, lines 78,79  page 4 lines 117-125 |
| Participants | 6 | **(*a*)**  ***Case-control study*—Give the eligibility criteria, and the sources and methods of case ascertainment and control selection. Give the rationale for the choice of cases and controls**  page 4 lines 117-125  ***Cross-sectional study*—Give the eligibility criteria, and the sources and methods of selection of participants**  To assess the association between hookworm and bone marrow iron deficiency, hookworm prevalence was compared between iron deficient and replete cases. .  page 4, lines 136 |
|  |  | **(*b*)**  ***Cohort study*—For matched studies, give matching criteria and number of exposed and unexposed** n/a  ***Case-control study*—For matched studies, give matching criteria and the number of controls per case** n/a |
| Variables | 7 | **Clearly define all outcomes, exposures, predictors, potential confounders, and effect modifiers. Give diagnostic criteria, if applicable**  page 6, lines 177-200  figure legends figure 1 and 2 main article |
| Data sources/ measurement | 8* | **For each variable of interest, give sources of data and details of methods of assessment (measurement). Describe comparability of assessment methods if there is more than one group. Give information separately for cases and controls in case-control studies and, if applicable, for exposed and unexposed groups in cohort and cross-sectional studies.**    page 4-5, lines 132-174  Ad 1, supporting information  Calis et al NEJM 2008 |
| Bias | 9 | **Describe any efforts to address potential sources of bias**  page 4, lines 122-125  Ad 2, supporting information |
| Study size | 10 | **Explain how the study size was arrived at**  figure S1  Ad 3, supporting information |
| Quantitative variables | 11 | **Explain how quantitative variables were handled in the analyses. If applicable, describe which groupings were chosen and why**  page 4-5  Ad 4, supporting information |
| Statistical methods | 12 | (*a*) **Describe all statistical methods, including those used to control for confounding**  page 6, lines 177-200 |
|  |  | (*b*) **Describe any methods used to examine subgroups and interactions**  page 6, lines 191-193 and 203-209 |
|  |  | (*c*) **Explain how missing data were addressed**  Ad 5, supporting information  Calis et al NEJM 2008 |
|  |  | (*d*) ***Cohort study*—If applicable, explain how loss to follow-up was addressed** n/a  ***Case-control study*—If applicable, explain how matching of cases and controls was addressed** n/a  ***Cross-sectional study*—If applicable, describe analytical methods taking account of sampling strategy n/a** |
|  |  | (*e*) **Describe any sensitivity analyses n/a** |

Continued on next page

| Results | | |
| --- | --- | --- |
| Participants | 13* | **(a) Report numbers of individuals at each stage of study—e.g. numbers potentially eligible, examined for eligibility, confirmed eligible, included in the study, completing follow-up, and analysed :** see figure S1 |
|  |  | **(b) Give reasons for non-participation at each stage:** see figure S1 |
|  |  | **(c) Consider use of a flow diagram** see figure S1 |
| Descriptive data | 14* | 1. **Give characteristics of study participants (e.g. demographic, clinical, social) and information on exposures and potential confounders**   table 1 and 2 main article  table S1  Calis et al NEJM 2008 |
|  |  | **(b) Indicate number of participants with missing data for each variable of interest:**  table 1 and 2 main article  table S1  table S2 |
|  |  | (**c) *Cohort study*—Summarise follow-up time (eg, average and total amount)** n/a |
| Outcome data | 15* | ***Cohort study*—Report numbers of outcome events or summary measures over time** n/a |
|  |  | ***Case-control study—*Report numbers in each exposure category, or summary measures of exposure:** table 1 main article |
|  |  | ***Cross-sectional study—*Report numbers of outcome events or summary** **measures**: see table 2 main article  table S1 |
| Main results | 16 | **Give unadjusted estimates and, if applicable, confounder-adjusted estimates and their precision (eg, 95% confidence interval). Make clear which confounders were adjusted for and why they were included.**  table S3  table S4 |
|  |  | **(*b*) Report category boundaries when continuous variables were categorized**  page 4-5  page 7, lines 212-217 and 232-233  figure legends figure 1 and 2 main article |
|  |  | **(*c*) If relevant, consider translating estimates of relative risk into absolute risk for a meaningful time period** n/a |
| Other analyses | 17 | **Report other analyses done—eg analyses of subgroups and interactions, and sensitivity analyses** n/a |
| Discussion | | |
| Key results | 18 | **Summarise key results with reference to study objectives**  page 8, lines 253-260 |
| Limitations | 19 | **Discuss limitations of the study, taking into account sources of potential bias or imprecision. Discuss both direction and magnitude of any potential bias**  page 10, lines 327-339 |
| Interpretation | 20 | **Give a cautious overall interpretation of results considering objectives, limitations, multiplicity of analyses, results from similar studies, and other relevant evidence**  page 8,9,10 |
| Generalizability | 21 | **Discuss the generalizability (external validity) of the study results**  page 9, lines 283-291 |
| Other information | | |
| Funding | 22 | **Give the source of funding and the role of the funders for the present study and, if applicable, for the original study on which the present article is based**  See ad 6 supporting information |

**Note:** An Explanation and Elaboration article discusses each checklist item and gives methodological background and published examples of transparent reporting. The STROBE checklist is best used in conjunction with this article (freely available on the Web sites of PLoS Medicine at http://www.plosmedicine.org/, Annals of Internal Medicine at http://www.annals.org/, and Epidemiology at http://www.epidem.com/). Information on the STROBE Initiative is available at www.strobe-statement.org.
