# Supplementary material for: Real-time PCR Demonstrates Ancylostoma duodenale Is a Key Factor in the Etiology of Severe Anemia and Iron Deficiency in Malawian Pre-school Children
Source: PLoS Negl Trop Dis. 2012 Mar 6;6(3):e1555. doi: 10.1371/journal.pntd.0001555 (PMC3295794; doi:10.1371/journal.pntd.0001555)
Supplement: Supplementary file 6 [file pntd.0001555.s001.doc]

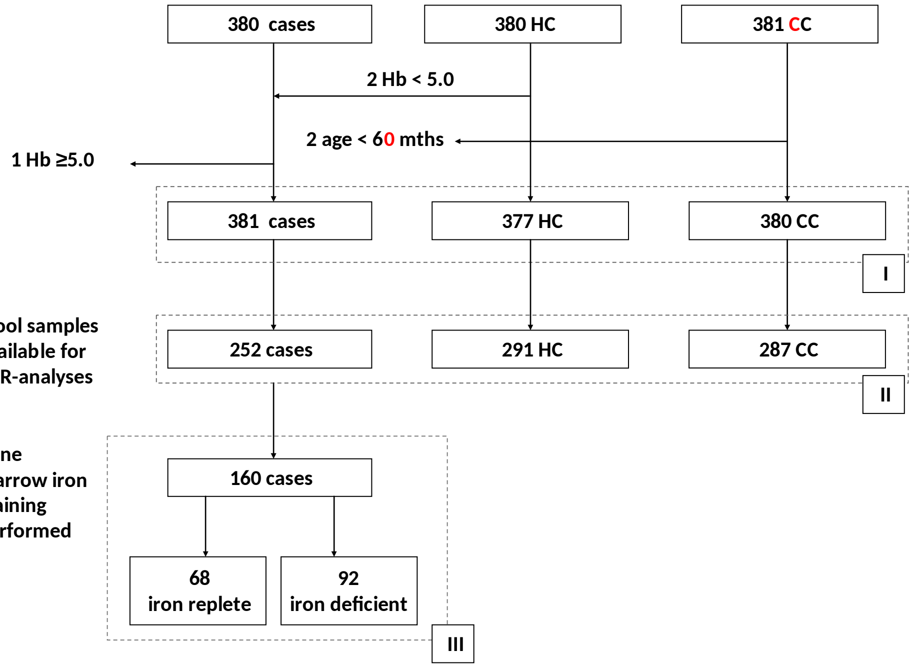
380 cases
380 HC
381 CC
2 Hb < 5.0
2 age < 60 mths
1 Hb ≥5.0
381 cases
377 HC
380 CC
I
Stool samples available for PCR-analyses
252 cases
291 HC
287 CC
II
Bone marrow iron staining performed
160 cases
68 iron replete
92 iron deficient
III
